# Biomechanical analysis of a synthetic femoral spiral fracture model: Do end caps improve retrograde flexible intramedullary nail fixation?

**DOI:** 10.1186/1749-799X-6-46

**Published:** 2011-09-18

**Authors:** Martin M Kaiser, Gregor Zachert, Robert Wendlandt, Marion Rapp, Rebecca Eggert, Christine Stratmann, Lucas M Wessel, Arndt P Schulz, Benjamin J Kienast

**Affiliations:** 1 Department of Paediatric Surgery, Medical Faculty of the University of Luebeck, Ratzeburger Allee 160, Luebeck, 23562, Germany; 2 Department of Biomechatronics and Academic Orthopaedics, Medical Faculty of the University of Luebeck, Ratzeburger Allee 160, Luebeck, 23562, Germany; 3 Department of Child and Adolescent Health, Medical Faculty of the University of Luebeck, Ratzeburger Allee 160, Luebeck, 23562, Germany; 4 Department of Paediatric Surgery, University of Mannheim, Theodor-Kutzer-Ufer 1-3, Mannheim, 68167, Germany; 5 Department of Traumatology, Orthopaedics and Sports Medicine, Trauma Center Hamburg, Bergedorfer Str. 10, Hamburg, 21033, Germany

**Keywords:** Elastic stable intramedullary nailing, ESIN, Flexible intramedullary nails, biomechanical testing, femoral shaft fracture, End Caps, Adolescents, Children

## Abstract

**Background:**

Elastic Stable intramedullary Nailing (ESIN) of dislocated diaphyseal femur fractures has become an accepted method for the treatment in children and adolescents with open physis. Studies focused on complications of this technique showed problems regarding stability, usually in complex fracture types such as spiral fractures and in older children weighing > 40 kg. Biomechanical in vitro testing was performed to evaluate the stability of simulated spiral femoral fractures after retrograde flexible titanium intramedullary nail fixation with and without End caps.

**Methods:**

Eight synthetic adolescent-size femoral bone models (Sawbones^® ^with a medullar canal of 10 mm and a spiral fracture of 100 mm length identically sawn by the manufacturer) were used for each group. Both groups underwent retrograde fixation with two 3.5 mm Titanium C-shaped nails inserted from medial and lateral entry portals. In the End Cap group the ends of the nails of the eight specimens were covered with End Caps (Synthes Company, Oberdorf, Switzerland) at the distal entry.

**Results:**

Beside posterior-anterior stress (4.11 Nm/mm vs. 1.78 Nm/mm, p < 0.001), the use of End Caps demonstrated no higher stability in 4-point bending compared to the group without End Caps (anterior-posterior bending 0.27 Nm/mm vs. 0.77 Nm/mm, p < 0.001; medial-lateral bending 0.8 Nm/mm vs. 1.10 Nm/mm, p < 0.01; lateral-medial bending 0.53 Nm/mm vs. 0.86 Nm/mm, p < 0.001) as well as during internal rotation (0.11 Nm/° vs. 0.14 Nm/°, p < 0.05). During compression in 9°- position and external rotation there was no statistical significant difference (0.37 Nm/° vs. 0.32 Nm/°, p = 0.13 and 1.29 mm vs. 2.18 mm, p = 0.20, respectively) compared to the "classic" 2-C-shaped osteosynthesis without End Caps.

**Conclusion:**

In this biomechanical study the use of End Caps did not improve the stability of the intramedullary flexible nail osteosynthesis.

## Background

Several treatment options for femoral shaft fractures in children and adolescents have been described. Children below the age of 3 can be treated with cast or extensional devices. In the past two decades the management of displaced femoral shaft fractures in older children has gradually evolved toward a more operative approach due to a more rapid recovery, faster reintegration of the patients and possible negative effects of immobilisation even in children [1,2]. Published complications of external fixation include rotational malalignment, secondary varus deformity as well as Re-Fractures or fractures in the area of the Pin entry [3-6]. Therefore, elastic stable intramedullary nail fixation (ESIN) of diaphyseal femoral fractures has become the most accepted method of treatment for children older than 3 years [7]. Contradictory information regarding the results can be found. Several retrospective studies report about a few or no complications [8-11]. Some authors report about skin problems and soft tissue irritation [12,13], while studies focused on complications following ESIN demonstrate problems between 10 and 50% [13-19]. In Ho's publication (94 fractures) the complication rate was 17% with 8 patients (significantly higher for patients aged 10 years or older) requiring an unplanned revision; average time to full weight bearing was 10 weeks and time to return to preoperative level of activity averaged 4.9 months [20]. Narayanan reported 41 soft-tissue problems, eight malalignments, two re-fractures and nine reoperations in 78 patients [18]. The highest number of complications is observed in complex fracture types and older children weighing more than 40 kg [17,20,21]. Due to instability some authors use an additional immobilization, additional screws or an additional external Fixateur [2,12,14,22-27]. Sink et al. changed their treatment concept towards submuscular plating, Kraus et al. recommend the external Fixateur for these fractures [28,29]. Our own retrospective data [30] revealed 43 children with closed fractures of the femur shaft between March 2002 and April 2007. 31 of these patients were treated with elastic stable intramedullary nailing (including three additional casts). Besides three cases of additional secondary immobilization eight of them needed reoperation: four patients due to varus deformity and four patients due to shortening of the fracture ("telescoping").

Due to our own mediocre results and the complications described in the literature we searched for an improvement of the method. Thus, the aim of our project was to determine, if the stability of the C-shaped osteosynthesis would be improved by different modifications [31]. The German guidelines for paediatric surgery also recommend the use of End Caps. They should improve stability in cases of instability following elastic stable intramedullary nailing [32] by interlocking the nails and preventing the "backing out". Despite that, very little clinical research has been published and proved the advantage of using these Caps [33]. In this second part of our project, we present the results of additional End Caps in composite bones using a spiral fracture type.

## Methods

**Figure 1:**
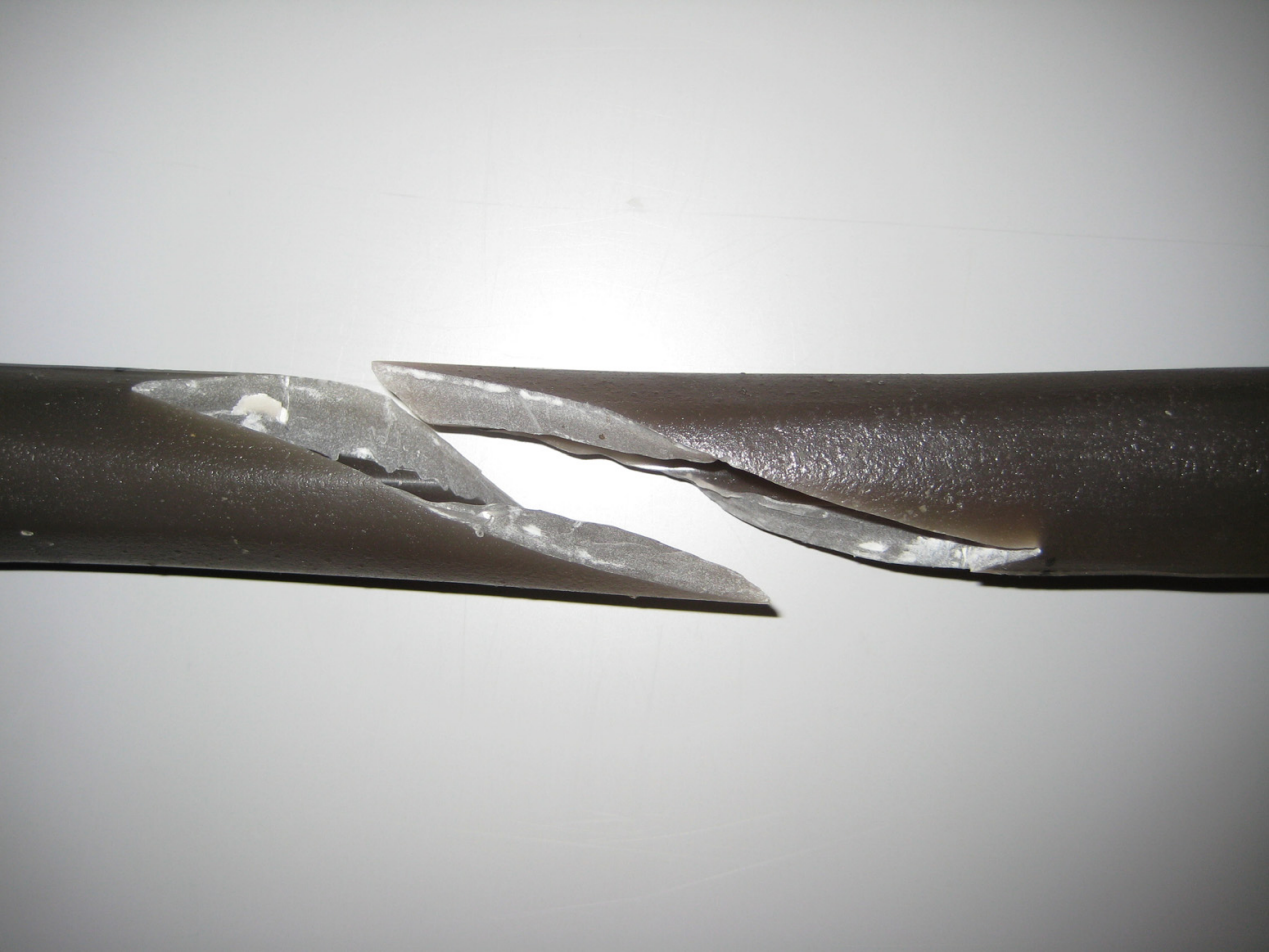
**Standard Sawbone^® ^Spiral Fracture**.

**Figure 2:**
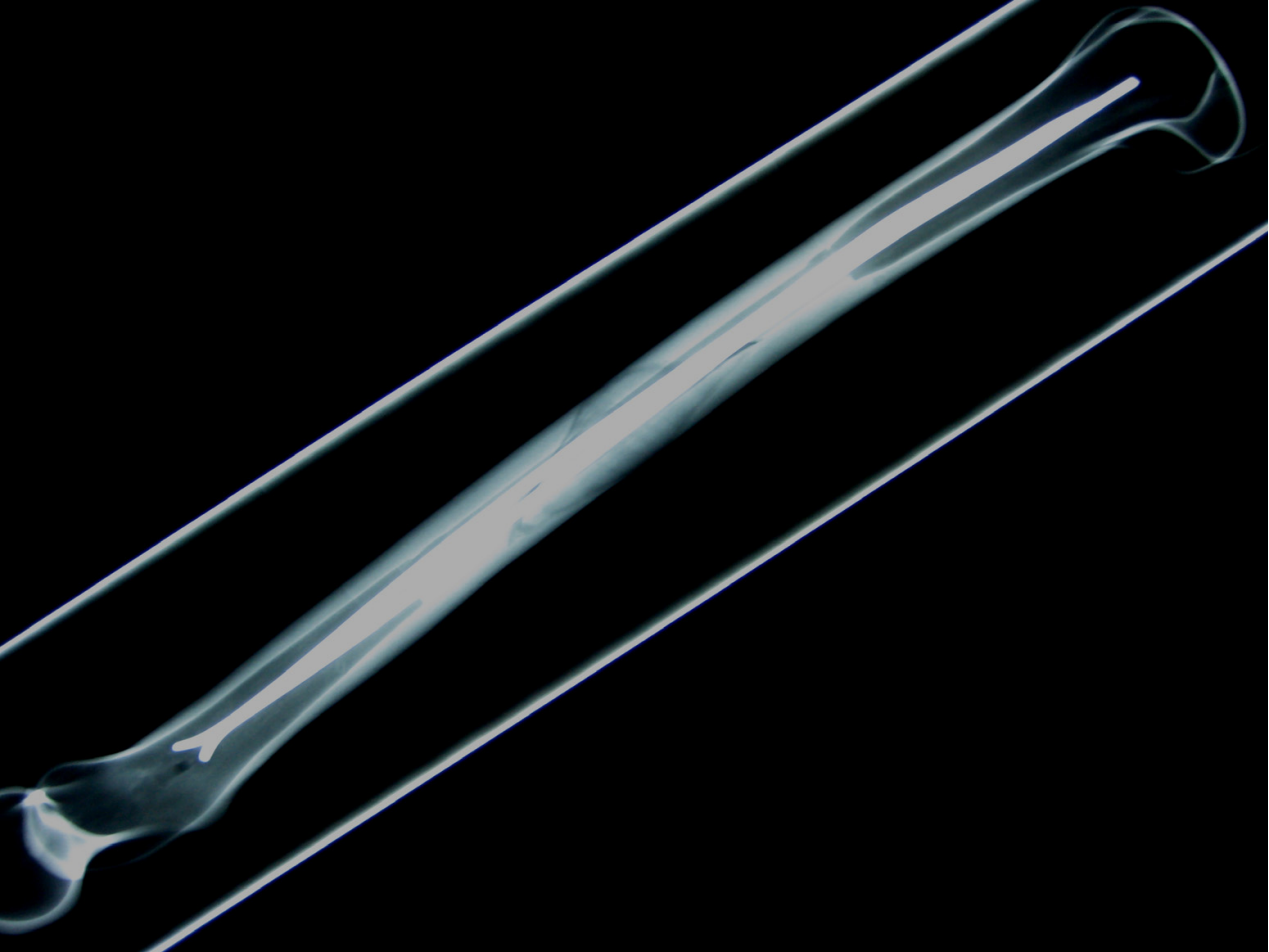
**Lateral Fluoroscopic image of a Sawbone^®^composite graft with a long spiral fracture after implantation of two elastic stable intramedullary nails; the endings of the nails (2 C-configuration) are inferior to the greater Trochanter**.

**Figure 3:**
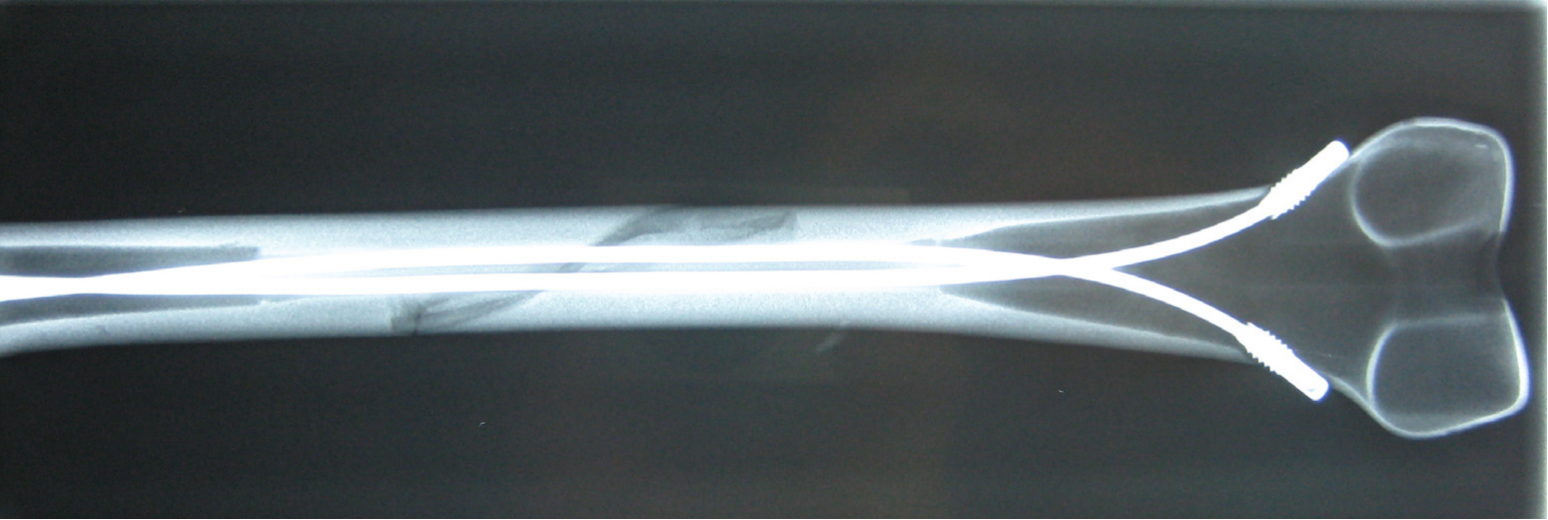
**AP Fluoroscopic image of a Sawbone^®^composite graft with a long spiral fracture after implantation of two elastic stable intramedullary nails with End Caps**.

**Figure 4:**
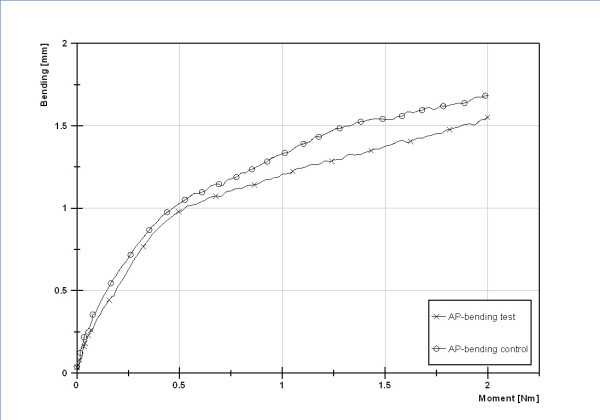
**Control cycle of testing to check for possible destructive changes which could have influenced the results**.

Mechanical testing was performed using 16 synthetic adolescent-sized composite femoral models (4^th ^generation, Sawbones^®^, Vashon, Washington, USA, European department in Sweden) that simulated both cortical and cancellous bone. The femoral model measured 45 cm in length, with a central canal diameter of 10 mm. A standard spiral fracture was created on Sawbones^® ^with a length of 100 mm (Figure 1). Due to the reason that paediatric Sawbone^® ^models are not available we decided to use this specimen as this Sawbone^® ^is corresponding to an adolescent sized femur and the approached question is most relevant for children weighing more than 40 kg and adolescents [17,20,21]. We used an established procedure to create the spiral fractures: Each standard mid-shaft spiral fracture was industrially sawen by Sawbone^®^. The fractures were identical: fracture length 100 mm with almost identical spiral and fragment angles. The parameters of the fracture were measured before the Sawbones^® ^were used in the biomechanical model [31]. All further details of this setting are described in our publication concerning the influence of different nail materials [31]. According to the literature the entry portals medial and lateral at the distal femoral physis were created by drilling a hole in the femur 2 to 3 cm proximal to the physis [34]. All nails were equally prebent 40 degrees, which brought the curve of the bending in contact with the fracture zone [10,34]. Eight femur models underwent retrograde intramedullary fixation (2 C-shaped ESIN pattern = "classical configuration" = "2E") with two 3.5-mm Titanium nails (Santech Nord^®^, Germany) placed through two drill holes (5-mm drill) at the distal femoral metaphysis 2 cm above the virtual physis. The nails ended at the proximal end of the canal, just inferior to the greater Trochanter (Figure 2). Fluoroscopic imaging was performed on each specimen to confirm the correct configuration and position. The osteosynthesis of the other eight models were created in a similar fashion with 3.5 mm 40° prebent Titanium Nails (Santech Nord^®^, Germany) and cylindric hollow-threaded End Caps ("2EEC") were applied (Fa. Synthes, Oberdorf, Switzerland, Figure 3). The specimens were tested using the UTM (Universal Testing Machine) Zwick 1465 testing machine (Zwick^® ^Company, Ulm, Germany). Custom-fit moulds were produced to secure the head of the femur and the femoral condyles in the testing machine. Each specimen was placed in the machine for a 4-point bending test, a torsional test and finally a compression test in 9°-position. The first cycle of the four individual tests was used as preconditioning; data for evaluation was collected from three subsequent cycles. After the last cycle of testing (9°-position) all specimens were again tested during anterior-posterior stress to check for possible destructive changes which could have influenced the results (Figure 4). The results of these cycles confirmed that all tests were performed without destruction of the osteosynthesis and the specimens.

**Figure 5:**
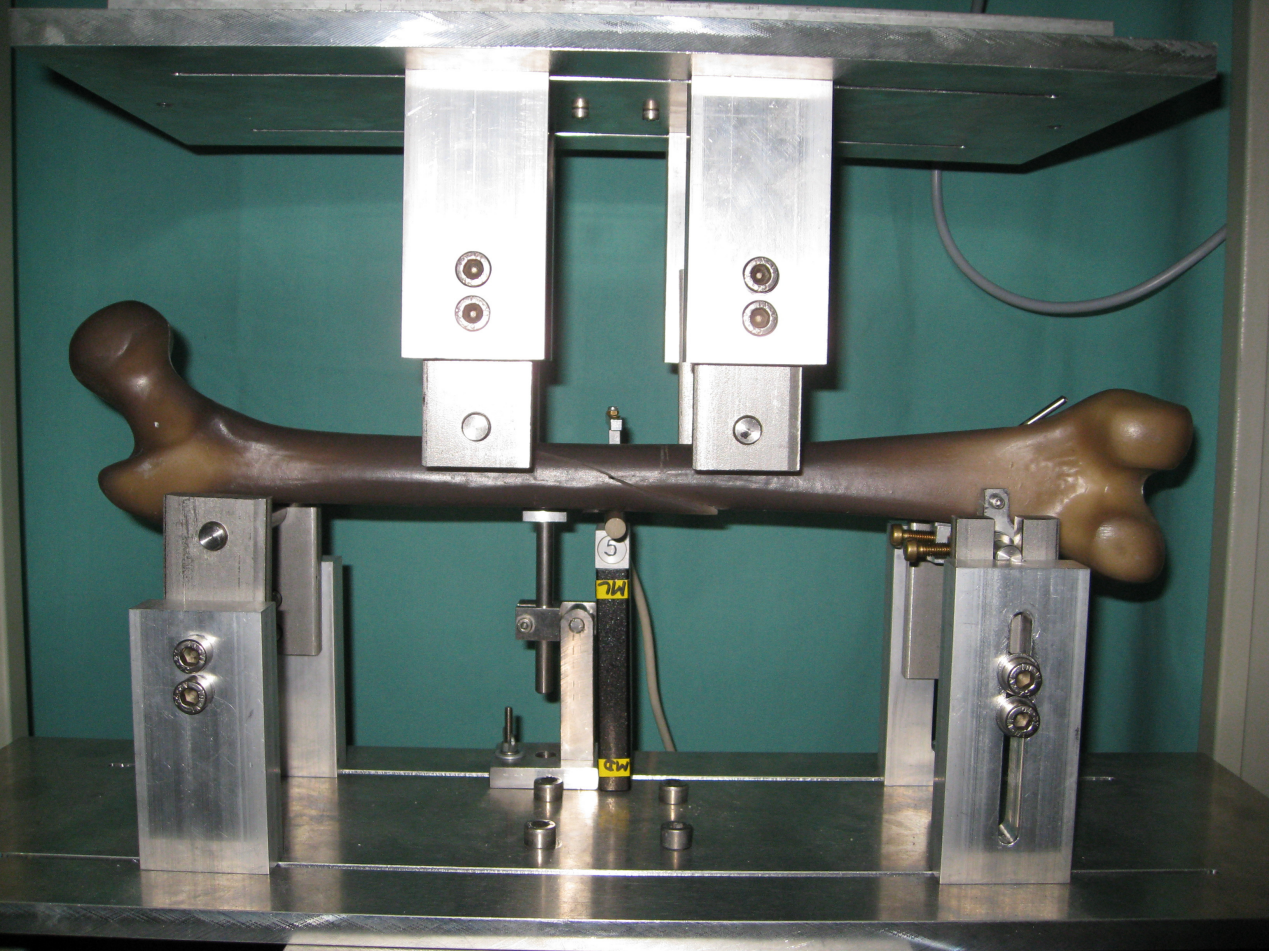
**Biomechanical testing of a Sawbone^® ^with spiral fracture in 4-point bending**.

The 4-point bending (Figure 5) was performed according to the ASTM F383-73 and F1264-03 description. With an incremental linear encoder bending was measured at a maximum of 5 Nm. Measurement took place at the midpoint of the two lower force bars, speed was set at 0,05 mm/s. Maximum bending was defined at 2 mm. After this was reached, tests were halted. The specimens were tested in the following order: anterior-posterior (AP), posterior-anterior (PA), lateral-medial (LM) and finally medial-lateral (ML). We chose fixed order to exclude any possible influence of random order on the results. For torsional testing the following criteria were set: The maximum allowed torsion during testing was 10°, the maximum torque was set at 10 Nm. Speed was set at 20°/min. With two angular encoders the torsion was measured. The femoral head area was gimbals-mounted. For compression testing the femur was positioned in 9° with a calibrated wedge ("AX9"). Fixation proximal and distal was performed with polymethylmetacrylate (PMMA, Technovit 4006) moulds for both sides. Acompression load up to 100 N was applied at a speed of 0.05 mm/s. Lateral shifting was measured at the Trochanter major, ventral shifting at the Crista intertrochanterica. Reduction of the fracture gap was measured using two incremental linear encoders (Product ID: MS30-1-LD-2, Megatron, Putzbrunn, Germany). Data (shortening in 9°-position, torsional stiffness in IR/ER and bending moments in 4-point bending) were analysed with SPSS 17.0 (SPSS Inc., Chicago, USA). Distributions were checked for normality (Shapiro-Wilk-Test) before statistical analysis was performed. Where significant departure from a normal distribution occurred a comparison of configurations regarding the evaluated parameters was performed with the Mann-Whitney-Test. If no significant departure from normal distribution was found, the F-Test and analyses of variance (ANOVA) were used. For adjusting significance levels to account for multiple comparisons post hoc pair comparison of homogenous distribution according to Scheffé and of inhomogeneous variances testing according to Games-Howell were parts of the control. All values are presented as mean values. Significance was set at p < 0.05.

## Results

All results of the stiffness of the two different configurations (2E = "classical configuration" vs. 2EEC = "classical configuration" with End Caps) are shown in Table 1. The 4-point bending tests from anterior-posterior showed mean values of the stiffness for the 2-C shaped ESIN configuration of 0.27 Nm/mm with End Caps (2EEC) compared to 0.77 Nm/mm for 2 Nails without End Caps (2E). Two nails were significantly more stable than the configuration with End Caps (p < 0.001). During the 4-point bending tests from posterior-anterior mean values of the stiffness for the 2-C shaped ESIN configuration of 4.11 Nm/mm with End Caps (2EEC) and 1.78 Nm/mm without End Caps (2E). In this testing ESIN with End Caps was significantly more stable than the classical setting (p < 0.001). During varus stress testing (medial-lateral direction) mean values were lower with End Caps (2EEC) than without (0.80 Nm/mm 2EEC vs. 1.10 Nm/mm 2E, p < 0.01). A comparable results was found for the 4-point bending tests from lateral-medial: mean values for the 2-C shaped ESIN configuration were 0.53 Nm/mm with End Caps (2EEC) and 0.86 Nm/mm without End Caps (p < 0.001). During torsional testing, the distal part of the femur was rotated 10° against the proximal part. As this occurred, the torque was determined. The internal rotation testing showed mean values of stiffness for the 2-C shaped ESIN configuration of 0.11 Nm/° with End Caps (2EEC) and 0.14 Nm/° without End Caps (2E). Thus, ESIN with End Caps was significantly less stable (p < 0.05) than the classical 2-C-shaped configuration. During external rotation testing no significant difference could be detected (0.37 Nm/° 2EEC vs. 0.32 Nm/° 2C; p = 0.14). Finally axial compression in 9°-position was measured in mm the level of the greater trochanter. Mean value was 1.29 mm with End Caps (2EEC) and 2.18 mm without End Caps (2C). By this, there was also no significant difference (p = 0.20).

**Table 1:** Summary of the results 2 ESIN vs. 2 ESIN with End Caps

	**2 Titanium Nails (2E)**		**2 Titanium Nails with End Caps (2EEC)**	**p-value**
	**Mean value (SD)**		**Mean value (SD)**	
				
**2 ESIN with End Caps *more *stable than 2 ESIN**				
				
Posterior-anterior	1.78 (1.31) Nm/mm	**<**	4.11 (2.24) Nm/mm	< 0.001
				
**2 ESIN with End Caps *less *stable than 2 ESIN**				
				
Anterior-posterior	0.77 (0.29) Nm/mm	**>**	0.27 (0.08) Nm/mm	< 0.001
Medial-lateral	1.10 (0.40) Nm/mm	**>**	0.80 (0.35) Nm/mm	< 0.01
Lateral-medial	0.86 (0.33) Nm/mm	**>**	0.53 (0.13) Nm/mm	< 0.001
Internal Rotation	0.14 (0.04) Nm/°	**>**	0.11 (0.01) Nm/°	< 0.05
				
**No statistical significant difference**				
				
External Rotation	0.32 (0.18) Nm/°	**~**	0,37 (0.11) Nm/°	0.13
Compression in 9°-Position	2.18 (1.37) mm	**~**	1.29 (1.61) mm	0.20

After the complete testing a second circle of anterior-posterior testing was done as a control.

Results of the first cycle compared to the control series showed no significant difference for 2-Nail-setup (p = 0.71) and the 2-Nail-configuration with End Caps (p = 0.78).

### Summary of Tests

With the use of End Caps (2EEC) a significantly higher stability could only be gained in stress tests from posterior-anterior. The classical setting with two elastic stable nails alone (2E) was more stable in bending from anterior-posterior, medial-lateral (Varus stress) as well as from lateral-medial (Valgus stress) and Internal rotation. No statistical significant difference could be found for External rotation and the compression in 9°-position.

## Discussion

This biomechanical study is the first published survey to deal with the influence of End Caps in the use of flexible nails for femoral shaft spiral fractures. Limitations of this study include the use of a synthetic bone model that possibly cannot precisely reproduce all in-vivo conditions. However, the synthetic bone model has been used successfully in previous biomechanical studies and provides more consistency among specimens than cadaveric bones [35-38]. Due to the configuration, the end of the nails could not be placed as proximal as it would be aspired at the operation in humans. This should be equalized as both configurations were established identically. During setup, the focus was on an identical surgical technique with an exact and even pre-bending and introduction of the nails. Improper location of the bends in the nails or the nails themselves may create an imbalance in the bending forces, which will result in an angular deformity. This technical mistake has been reported in the literature [10]. By this means the proper configuration of the nails was achieved more precisely than in a real surgical situation. Despite that, we saw some difference between the eight nail configurations of each group. We believe that this is due to slight differences at the fracture site despite industrial production. In oblique fractures these differences are expected to be much smaller, because even during industrial production a transverse or an oblique fracture is much easier created than a more complex spiroid type fracture. The biomechanical properties of retrograde C-shaped flexible intramedullary nailing have been described in the literature [39-46]. Most of the authors studied oblique or transverse fractures; only two studies examined the spiral type fracture [45,46]. More or less comparable data of biomechanical testing is thereby only available in these studies. In an evaluation of spiral fractures in 10 canine bones Benz et al showed that stabilization with intramedullary flexible nails was only possible in 3 cases. In the other cases the osteosynthesis did not even gain sufficient stability to make testing setup possible. Gwyn et al performed biomechanical testing with different fracture types in synthetic bone models using 2 titanium elastic nails of 4 mm diameter to evaluate the femoral stability with intramedullary nails. Only external and internal rotation forces were tested. In this study, transverse and comminuted fractures were the least stable. For spiral fracture types, stability was much lower in internal rotation (our data: 0.11 Nm/°) compared to external rotation (our data: 0.37 Nm/°). The reason for this difference is the direction of the spiral fracture - one direction will lead to a slipping of the fracture edges while during movement in the other direction the edges will be caught. In transverse or oblique fractures the internal and external rotational forces are more or less equal. These results show that a stabilization of complex fractures is possible- but very unpredictable in terms of the stability gained with different fracture types and acting forces. It is an interesting point that other study groups decided to test only one or two allocation levels. In all of these studies no rational was given for this [39,42,44,45]. In contrast, we are certain that the complex structure of a spiral-fracture requires testing in all levels. We detected different results concerning stability: more stability in the posterior-anterior bending with End Caps vs. less stability in anterior-posterior-/medial-lateral- and lateral-medial-bending as well as during Internal rotation.

In summary, we could not find a benefit in adding End Caps to the classical way of elastic stable intramedullary nailing in our in vitro synthetic model of spiral femoral fractures. The technique could not provide a more stable fixation to maintain length and rotational control of these spiral midshaft fractures. The only advantage was seen in posterior-anterior bending.

This is in contrast to the published data of Anastasopoulos et al, were 7 patients with diaphyseal femoral fractures (classified as "oblique or comminutive", without explicit data on age and body weight) and three patients with tibia fractures were operated with the use of End Caps. Concerning only the femoral fractures, difficulties were encountered in two patients while inserting the End Caps: in one case it was impossible to screw the End Cap into the bone cortex and in the second the caps were held rather loosely in the bone. In conclusion, fitting of the End Caps was quoted as "fair", because in 6 cases the end of the nail was not 100% in contact with the end cap. They described only one 5-10 mm shortening, one 10-mm leg shortening in another patient in whom the end caps could not be properly inserted and one Internal rotation greater than 10°. One patient gained an additional immobilisation due to pain, another due to important knee instability with a patellar fracture. No weight bearing was allowed for at least three weeks. The authors pointed out, that removing the implants was eased by the use of the End Caps after bone healing [33]. The solution might be less than 100% contact of the nails in the End Caps: too close contact might lead to a small, almost invisible distraction at the fracture site with consecutive loss of stiffness in a model without surrounding periosteum and other soft tissue.

For the future further biomechanical research is required to improve this type of osteosynthesis and to make it more feasible for different types of fractures. Also transverse and oblique fractures need to be tested with the combination of elastic stable intramedullary nailing and End Caps.

## Competing interests

All authors declare that no benefits in any form have been received or will be received from a commercial party related directly or indirectly to the subject of this article. The elastic stable nails used in our testings were sponsored by Santech Nord Company, Schneverdingen, Germany.

## Authors' contributions

MMK is the responsible author and the head of the study group. GZ and RW are responsible for all testings in the laboratory and edited/reviewed the manuscript. RE, CS and APS did the testings and edited/reviewed the manuscript. LMW was responsible for the statistics. MR was responsible for translation and proof-reading of the manuscript. BJK was responsible for translation, proof-reading, and supervision of all versions of the manuscript. All authors read and approved the final manuscript.
